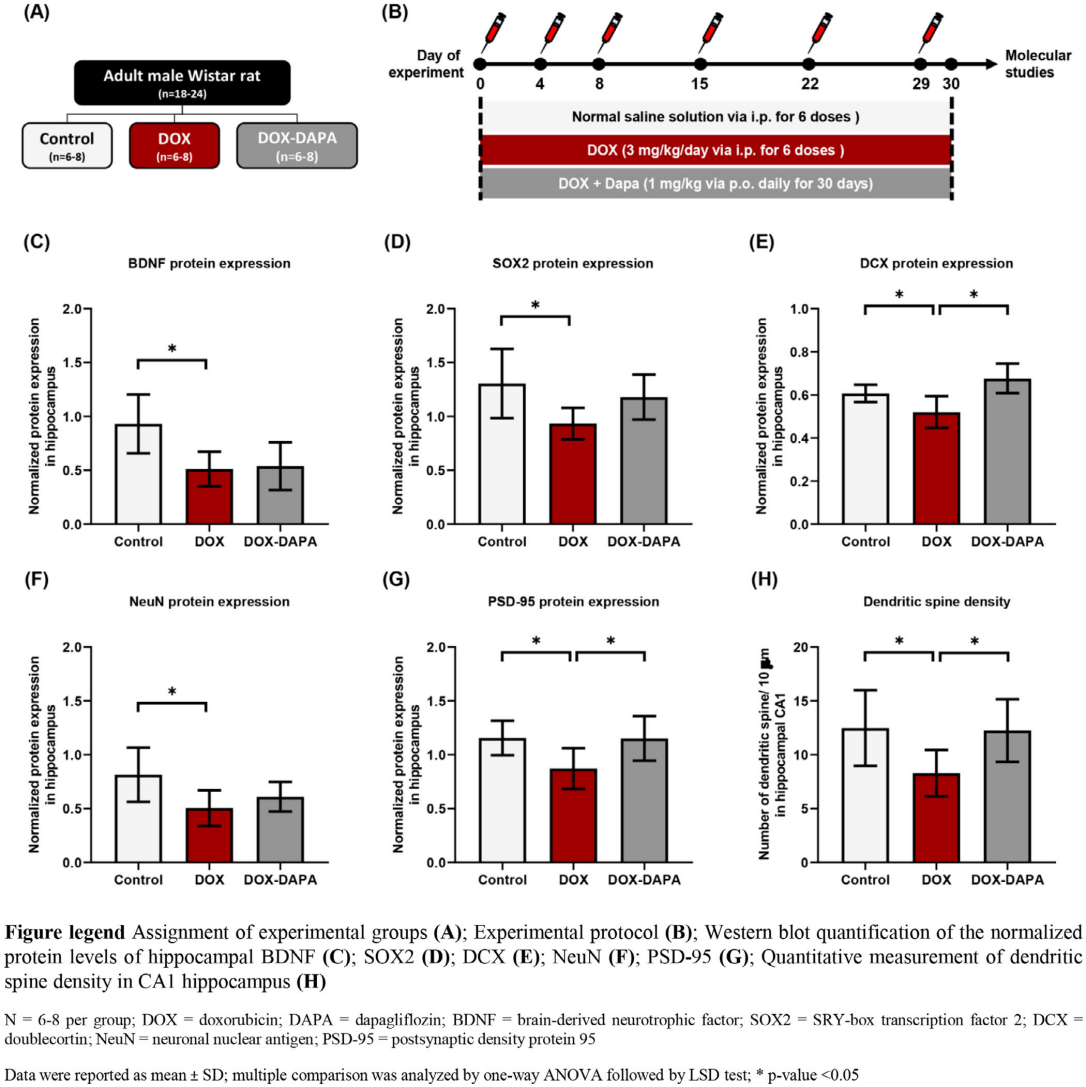# Neuroprotective Effects of Dapagliflozin on Hippocampal Adult Neurogenesis and Synaptic Integrity in Rats with Doxorubicin‐induced Chemobrain

**DOI:** 10.1002/alz70855_100062

**Published:** 2025-12-27

**Authors:** Patcharapong Pantiya, Titikorn Chunchai, Hiranya Pintana, Chayadom Maneechote, Busarin Arunsak, Nipon Chattipakorn, Siriporn C Chattipakorn

**Affiliations:** ^1^ Neurophysiology Unit, Cardiac Electrophysiology Research and Training Center, Faculty of Medicine, Chiang Mai University, Chiang Mai, Thailand; ^2^ Center of Excellence in Cardiac Electrophysiology Research, Chiang Mai University, Chiang Mai, Thailand; ^3^ Cardiac Electrophysiology Unit, Department of Physiology, Faculty of Medicine, Chiang Mai University, Chiang Mai, Thailand; ^4^ Department of Oral Biology and Diagnostic Sciences, Faculty of Dentistry, Chiang Mai University, Chiang Mai, Thailand

## Abstract

**Background:**

Doxorubicin, a chemotherapeutic agent, frequently causes debilitating neurological deficits known as chemobrain, as indicated by impaired neurogenesis and synaptic dysplasticity (1). Sodium‐glucose co‐transporter 2 (SGLT2) inhibitors, including dapagliflozin, also have neuroprotective properties beyond their anti‐diabetic activity (2). However, the effects of dapagliflozin on chemobrain following doxorubicin treatment remain unexplored. Therefore, this study aimed to investigate the neuroprotective effects of dapagliflozin in rats with doxorubicin‐induced chemobrain.

**Method:**

Adult male Wistar rats were divided into three groups: (1) Control (NSS), (2) DOX (doxorubicin; 3 mg/kg/day administered intraperitoneally for 6 doses over 30 days), and (3) DOX‐DAPA (combined treatment with 3 mg/kg/day doxorubicin administered intraperitoneally for 6 doses and 1 mg/kg dapagliflozin administered orally for 30 days). At the end of the experiment, the rats were euthanized for molecular analysis in the hippocampus. The experimental protocol is summarized in Figures A and B.

**Result:**

We found that DOX‐treated rats developed chemobrain in the hippocampus, including abnormal adult neurogenesis, as indicated by decreased BDNF, SOX2, DCX, NeuN, and PSD‐95 protein expression, and synaptic dysplasticity, as shown by a reduction in dendritic spine density, compared to the control group (Figures C‐I). Interestingly, dapagliflozin co‐treatment exhibited neuroprotective effects in DOX‐treated rats, as shown by increased DCX and PSD‐95 protein expression and dendritic spine density in the hippocampus, compared with DOX‐treated rats (Figures C‐I).

**Conclusion:**

Our findings indicate that doxorubicin induces chemobrain, as shown by impairments in adult neurogenesis and synaptic integrity. Dapagliflozin mitigates the chemobrain induced by doxorubicin. Thus, dapagliflozin may serve as a therapeutic approach to alleviate chemobrain induced by doxorubicin.